# Exploring the experiences of adults with stroke in virtual community-based stroke programs: a qualitative descriptive study

**DOI:** 10.1186/s12913-024-11043-7

**Published:** 2024-05-07

**Authors:** Amy Cruickshank, Emma D’Andrea Brooks, Christina Sperling, Michelle LA Nelson, Hardeep Singh

**Affiliations:** 1 Department of Occupational Science & Occupational Therapy, Faculty of Medicine, University of Toronto, 500 University Ave, Toronto, ON M5G 1V7 Canada; 2 March of Dimes Canada, 202-885 Don Mills Rd., Toronto, ON M3C 1V9 Canada; 3 Lunenfeld-Tanenbaum Research Institute, Sinai Health System, 1 Bridgepoint Dr, Toronto, ON M4M 2B5 Canada; 4 Institute of Health Policy Management and Evaluation, Dalla Lana School of Public Health, University of Toronto, 155 College St 4th Floor, Toronto, ON M5T 3M6 Canada; 5 KITE Research Institute, Toronto Rehabilitation Institute-University Health Network, 520 Sutherland Dr, Toronto, ON M4G 3V9 Canada; 6 Rehabilitation Sciences Institute, Temerty Faculty of Medicine, University of Toronto, 500 University Ave, Toronto, ON M5G 1V7 Canada

**Keywords:** Stroke, Community-based organizations, Qualitative, Virtual

## Abstract

**Background:**

Stroke is among the top contributors to disability and can impact an individual’s cognition, physical functioning, and mental health. Since the COVID-19 pandemic, several community-based organizations have started delivering stroke programs virtually. However, participants’ experiences in these programs remain understudied, and evidence-based guidelines to inform and optimize virtual stroke program development and delivery are lacking. Thus, this study aimed to describe the perspectives and experiences of individuals with stroke who participated in virtual community-based organization stroke programs, including perceived access and participation facilitators and barriers and suggestions for improving these programs.

**Methods:**

A qualitative descriptive design was used to gather participant experiences through semi-structured interviews. Audio-recorded interviews were conducted on Zoom and transcribed verbatim. Adult participants who had experienced a stroke and attended at least one Canadian virtual community-based organization stroke program were recruited. Data were analyzed using inductive thematic analysis.

**Results:**

Twelve participants (32–69 years, 2–23 years post-stroke, eight women and four men) participated in this study. Five themes were identified: (1) motives to join virtual community-based organization stroke programs, including gaining peer connections, knowledge and information; (2) perceived barriers to accessing and participating in virtual community-based organization stroke programs, including technology inequities, difficulties navigating technology, and inadequate facilitation; (3) perceived facilitators to accessing and participating in virtual community-based organization stroke programs, including remote access, virtual platform features and program leader characteristics/skills; (4) unmet needs during virtual community-based organization stroke programs, including in-person connection and individualized support; and (5) suggestions and preferences for improving virtual community-based organization stroke programs, including program facilitation, content and format.

**Conclusions:**

Study findings highlight opportunities to improve virtual community-based organization stroke programs to optimize participant experiences and outcomes. Addressing the barriers and suggestions identified in this study may improve virtual community-based organization stroke programs’ access and quality.

**Supplementary Information:**

The online version contains supplementary material available at 10.1186/s12913-024-11043-7.

## Background

Individuals are living longer with stroke [1], and the severity of strokes has increased in the last ten years, intensifying the need for post-stroke services [2, 3]. Individuals with stroke may experience various impairments, including changes in speech/language, sensation, cognitive functioning, motor abilities and sensation [4–9]. Activities of daily living are often impacted [10–12], in addition to living with lasting consequences on one’s ability to drive [13], socialize [14–16] and re-engage in the workforce [17, 18], which can influence reintegration into the community [19–21]. Therefore, a need exists for continued care in the community to support individuals with stroke [19, 22, 23]. Community stroke services have been found to support stroke recovery by enhancing the individual’s social connections [24], reducing psychosocial distress [25, 26] and improving physical functioning [27]. The COVID-19 pandemic has changed how community services are delivered [28, 29].

Instigated by the COVID-19 pandemic, there has been an accelerated shift to providing virtual community-based stroke programs [28, 29] and, consequently, an emergence of research examining the outcomes of the newly delivered virtual programs [30–32]. For instance, a 2022 study by Gray & colleagues examined Choose to Move, a community-based physical activity program which transitioned to virtual programming during the COVID-19 pandemic [30]. They found that the virtual program was feasible, implemented satisfactorily, and remained true to program objectives [30]. Another study by Jennings & colleagues [32] described Gerofit, an exercise program offered to veterans and its transition to virtual services shortly after the start of the COVID-19 pandemic. Jennings and colleagues [32] demonstrated that virtual exercise classes could provide adequate intensity for health promotion, as suggested by most participants (*n* = 365) reporting that their exertion rating met the national recommendation. They also found that participants reported no adverse events in the first three months of virtual program implementation [32]. Similarly, Collins & Layne [31] examined the opportunities and challenges of an educational and exercise community-based fall prevention program for seniors that transitioned to a virtual format during the COVID-19 pandemic. Collins & Layne [31] highlight the need to understand the various opportunities and obstacles within virtual programs to improve program quality, as they found less participant engagement following the initial shift to virtual services due to barriers related to participants accessing the virtual content. Overall, virtual programs are acceptable and feasible [30–32] and demonstrate benefits to users, including removing travel/geographical barriers [30, 33]. However, given that the widespread use of virtual programs is recent, virtual stroke programs may require further research to optimize program delivery for individuals with stroke.

While virtual programs have multiple benefits [30, 33–35], the shift to virtual programming may present barriers to participation and engagement in virtual stroke programs for people with stroke [36]. For instance, the digital divide presents a barrier to virtual programs, particularly for older adults who may have greater difficulty accessing and using technology [30]. In addition, common participant-reported barriers in stroke telerehabilitation included troubleshooting technology when internet connectivity issues arose, difficulty using the technology, and lack of overall experience with technology [37–39]. While existing studies have evaluated virtual stroke programs using participant feedback [33, 40], a comprehensive synthesis of participant experiences within virtual stroke programs delivered by community-based organizations (CBOs) is lacking.

Due to the sudden nature of stroke [41] and limited active time spent in rehabilitation units [42, 43], CBOs are integral to stroke recovery following hospital discharge [22, 44]. In Canada, CBOs provide various stroke services in the community, including psychosocial, functional, physical, and informational [22]. Many CBO programs were delivered in person before the COVID-19 pandemic [22], and rapidly shifted to virtual during the pandemic [29, 45]. One study examined Moving On after Stroke, a virtual community-based program for individuals who had experienced a stroke and their caregivers [33]. Taylor and colleagues [33] found enablers and barriers to videoconferencing program participation and provided suggestions to develop the program. However, they acknowledged limitations such as only sampling participants who finished the program and having the program co-facilitator interview participants for the study, which introduced bias [33]. In addition, the experiences of those who did not continue with the program because of potential dissatisfaction or other barriers were not represented [33]. Another study examined participant and program provider feedback on a community-based memory program for stroke survivors that was delivered using videoconferencing [40]. While Lawson & colleagues [40] found that participants provided positive feedback on the program, all participants had completed the entire program and were required to be proficient with the videoconferencing tools. More research representing an increasingly diverse range of participant experiences, including those who may or may not have completed multiple virtual CBO stroke program sessions, is necessary to understand participation barriers and facilitators.

### Study aim

To inform virtual stroke program recommendations grounded in participant experiences, the current study aimed to describe the experiences and perceptions of individuals who participated in virtual CBO stroke programs on access and participation facilitators and barriers and their suggestions for improving these programs.

## Methods

### Design

This study followed a qualitative descriptive design [46], which is suitable for comprehensively examining understudied concepts from first-hand experiences [47] and enabled us to capture an in-depth understanding of participants’ perspectives of perceived facilitators and barriers in virtual stroke programs delivered by CBOs; hereafter referred to as virtual CBO stroke programs. Research ethics board approval was obtained from the University of Toronto Research Ethics Board (#41,969). All methods were carried out in accordance with relevant guidelines and regulations and informed consent was obtained from all participants. The Standards for Reporting Qualitative Research checklist [48], a qualitative research reporting checklist, was followed to improve the comprehensiveness and depth of our report.

### Participant recruitment and setting

Participants were recruited between January 10, 2022, and February 1, 2022, through advertisements shared on social media by the research team and Canadian CBOs providing virtual stroke programs, including March of Dimes Canada and the Evergreen Communication Therapy For Survivors. The advertisements instructed potential participants to contact the research team by email or phone. Participants who met the following inclusion criteria were recruited: (1) experienced any severity or type of stroke, (2) attended at least one virtual CBO stroke program session [22], and (3) were over 18 years of age. Thirteen individuals were recruited; one individual withdrew prior to data collection as they were no longer interested in participating in this study.

### Data collection

Twelve participants who experienced a stroke and attended a virtual CBO stroke program session at least once were interviewed between January and February 2022. Participant experiences were elicited through one semi-structured interview conducted by HS (woman, Assistant Professor, MScOT, PhD) over the Zoom video conferencing platform or by telephone. Participants were probed about their experiences with the virtual stroke program delivery, their motives for virtual participation, perceived facilitators and barriers to accessing and participating in the program, and any suggestions or expectations for future stroke programming using a semi-structured interview guide (see supplementary materials for interview guide). The interview guide was developed by HS, who has experience conducting qualitative interviews. The open-ended nature of semi-structured questions allowed researchers to gather comprehensive data by exploring participant responses further through a predetermined set of prompts and follow-up questions [49, 50] while allowing flexibility for other questions based on the participants’ story-telling of their experiences. Interview responses were recorded and transcribed verbatim on Zoom, followed by transcript verification by AC (woman, student occupational therapist) and EDB (woman, student occupational therapist) to ensure the accuracy of transcripts. Data were de-identified using participant codes and then stored on a server approved by the ethics board. Participant information was collected, such as age, sex, gender, ethnicity, location, post-stroke difficulties, years since the stroke, type of program attended, access to technology, and comfort with technology.

### Data analysis

The data management software NVivo version 12 was used to organize qualitative data to generate themes. Data were transcribed and then analyzed descriptively following an inductive thematic analysis following the procedures described here [51]. An inductive thematic analysis aligned with a qualitative descriptive approach as it enabled the generation of themes closely related to the data and would be reflective of participant perspectives [51]. The analysis began with AC and EDB listening to the audio recordings to understand the data. Next, AC and EDB independently created initial inductive codes for data on NVivo version 12, initially coding each line of two interview transcripts to produce a codebook, which was used to analyze the remaining interview transcripts. To ensure the trustworthiness of the study results, both researchers analyzed the data independently and then conducted investigator triangulation by collaboratively analyzing data to add multiple perspectives and conclusions [52]. In addition, the researchers adopted the principle of data saturation [53], wherein they determined that the data collected and analyzed was sufficient to address this research study’s aim and decided not to interview additional participants.

After the initial coding of all data, AC and EDB began to analyze codes by sorting them into various themes based on patterns within the data. For example, initial codes “personalized feedback” and “individualized support” were grouped into the subtheme “unmet need for individualized support” (subtheme 4b) within the broader theme “unmet needs during virtual CBO stroke programs” (theme 4). Tables and thematic maps were also created to visually explore potential relationships between codes and emerging themes and subthemes.

## Results

### Description of individual study participants and CBO stroke programs

Twelve individuals with stroke (average age 52.6 years) participated in this study, including eight women and four men. Participants had experienced a prior stroke between 2 and 23 years ago (average 6.2 years) (see Table 1 for participant details). Most participants reported having university-level education (*n* = 6, 50%), four (33%) had a college-level, and one (8%) had a grade school and one (8%) had a graduate-level education. Most participants were born in Canada (*n* = 8, 67%), while four (33%) were outside of Canada (i.e., Columbia, South Africa, Malaysia, and the United States). While English was not the first language of three participants (25%), only one participant reported challenges reading English. In terms of post-stroke impairments, participants reported a variety of challenges with walking/standing, communication (e.g., understanding someone, speaking, cognition, vision, upper extremity function, and fatigue.

All participants reported attending synchronous CBO stroke programs focusing on physical activity, aphasia, mental health, peer support, and stroke-related education. Participants indicated that the programs were primarily delivered on the Zoom video conferencing platform. In addition, some participants also accessed asynchronous stroke-related education and information through recorded webinars, emails, and chat boards. All participants owned or had access to a computer, smartphone, tablet and Internet connectivity. Participants were comfortable (*n* = 9, 75%) or somewhat comfortable (*n* = 2, 17%) using a computer, smartphone or tablet, and all but one participant reported using social media daily or almost daily.

**Table 1:** Participant demographics table

Participant Code	Age (Years)	Sex & Gender	Years Post-Stroke	Ethnicity
PV1	64	Female, Woman	3	Not reported
PV2	32	Female, Woman	2	Latin American
PV3	64	Male, Man	6	White-European
PV4	65	Male, Man	6	White
PV5	39	Female, Woman	2	South Asian
PV6	56	Female, Woman	3	White-North American
PV7	69	Female, Woman	2	White-European
PV8	50	Female, Woman	23	Chinese
PV10	40	Male, Man	2	White
PV11	33	Female, Woman	11	White
PV12	58	Female, Woman	3	White-European
PV13	61	Male, Man	11	White-European

Through analysis of the interview data, five broad themes were identified as presented in Table 2: (1) Motives to join virtual CBO stroke programs, (2) Perceived barriers to accessing and participating in virtual CBO stroke programs, (3) Perceived facilitators to accessing and participating in virtual CBO stroke programs, (4) Unmet needs during virtual CBO stroke programs, and (5) Suggestions and preferences for improving virtual CBO stroke programs.

**Table 2:** Themes, subthemes and supporting quotes

Themes	Subthemes	Supporting quotes
**Theme 1: Motives to join virtual CBO stroke programs**	*Subtheme 1a: Seeking peer connection* *Subtheme 1b: Gaining stroke-related knowledge and information*	“I was feeling a little bit like out on my own kind of thing, but then like I’m not on my own because I have all these other people who are who have gone through stroke and are trying to get back on their feet” (PV8, age 50, 23 years post-stroke). “What you have a chance to do is listen to areas that you’re unaware of, and of course, everyone’s stroke is very different right so. So you glean what you can from it, but then they provide information to other sources of information that you can find more specific things about so, and it’s really across the board, they cover so much, which is really good, and you can pick and choose which ones, you want to be involved in which is, which is great. But then they send out all of the information and the email telling -talking about the next one, so you get everything anyway, which is really good” (PV7, age 69, 2 years post-stroke).
**Theme 2: Perceived barriers to accessing and participating in virtual CBO stroke programs**	*Subtheme 2a: Technological inequities* *Subtheme 2b: Difficulties navigating technology* *Subtheme 2c: Impact of facilitator instruction*	“I had all new equipment and had to get it hooked up” (PV1, age 64, 3 years post-stroke). “The first thing is like the technical glitches. It kind of throws you because, you know, sometimes the internet is slow, you lose the picture, the connection to the person, and I think those are distracting those things are distracting” (PV5, age 39, 2 years post-stroke). “After I got on, you know, got the Zoom setup and got the technical part. I guess you could say the technical part of it was challenging. But I don’t know how you could ever change it” (PV1, age 64, 3 years post-stroke).
**Theme 3: Perceived facilitators to accessing and participating in virtual CBO stroke programs**	*Subtheme 3a: Remote access to virtual CBO stroke programs* *Subtheme 3b: Enabling virtual platform features* *Subtheme 3c: Program leader characteristics*	“I see huge benefits with the- with the online format, in that you get people from very diverse geographical areas participating” (PV3, age 64, 6 years post-stroke). “It also allows, you know, eliminates the need for transportation, so if, if you can’t drive I mean Zoom is amazing, you know it’s a great technology” (PV3, age 64, 6 years post-stroke). “[the program leader] has it in small groups so that you can actually leave your microphone on or you know turn it off and turn it on when necessary” (PV1, age 64, 3 years post-stroke). “ “I felt that he was a seasoned communicator and could deliver like he needed to deliver” (PV1, age 64, 3 years post-stroke).
**Theme 4: Unmet needs during virtual CBO stroke programs**	*Subtheme 4a: Unmet need for in-person connection* *Subtheme 4b Unmet need for individualized support*	“And then and then taking the time, especially with stroke patients to stop in and check in during that program, how are we doing, what are the questions you might have, Is anybody- is anybody finding this to a high level, is anybody find - You know just checking in, because we’re all at different stages within the stroke process and, and all have had different types of strokes” (PV7, 69, 2 years post-stroke). “When you’re working in a group, you always work to the lowest common denominator. And with the stroke victims, you have every kind and every ability represented. I just feel that we’re doing the exercises, but all of us are doing them at our own ability. But there’s nobody pushing you like, great you leaned over half an inch now. Do you think you can go a little bit further? Good, you know, or something” (PV1, age 64, 3 years post-stroke).
**Theme 5: Suggestions and preferences for improving virtual CBO stroke programs**	*Subtheme 5a: Suggestions for effective facilitation of virtual programs* *Subtheme 5b: Virtual content preferences* *Subtheme 5c: Delivery format preferences*	“I think when the online facilitator engages with all the people, one by one, each one individually, and they know that they counted [unintelligible] their product is meaningful to them so they know exactly what you need and so that that helps I think for the facilitator to know you more” (PV8, age 50, 23 years post-stroke). “Old fashioned compliments or encouragement. Myself, I know that encouragement is a really, really important part…” (PV1, age 64, 3 years post-stroke). “To find a group for a group of stroke survivors is very easy, but to find a group of stroke survivors that aren’t of the median age of 75 and up is very hard. My plate is different than their plate. My plate is to get back to work and have a semblance of a life again. Their plate is to be comfortable in their retirement” (PV10, age 40, 2 years post-stroke). “I would probably more lean towards the online just because it’s more convenient, especially, I have two young children” (PV10, age 40, 2 years post-stroke).

### Theme 1: motives to join virtual CBO stroke programs

The first theme describes participants’ reasoning for participating in virtual CBO stroke programs. Participants commonly reported two motives for joining virtual CBO stroke programs: seeking peer connection (subtheme 1a) and gaining knowledge and information (subtheme 1b).

#### Subtheme 1a: seeking peer connection

A commonly cited motive for joining a virtual CBO stroke program was to connect with others who have experienced a stroke. PV3 emphasized the importance of meeting others who had gone through similar experiences with their stroke: “…when you speak to other persons who have gone through the same thing, it gives you a different lens than if you’re speaking to a strictly a health care provider” (PV3, age 64, 6 years post-stroke). The importance of peer connection for building community was also a motivating factor for involvement in virtual programs, with another participant stating,

It was comforting because…there is a sense of loneliness you feel, and then when you speak to someone that kind of knows or understands it, it kind of reassures you that you’re not crazy, and it also reassures you that it’s normal what you’re going through and there’s other people out there (PV5, age 39, 2 years post-stroke).

Notably, during the COVID-19 pandemic, participants described virtual CBO stroke programs as one of the few sources of social interaction, with one participant stating, “…you can’t really go anywhere with COVID. So it was nice to have some connections that I could make…I’ve gotten to know quite a few people” (PV4, age 65, 6 years post-stroke).

#### Subtheme 1b: gaining stroke-related knowledge and information

The second motive participants described that fueled their desire to join a virtual CBO stroke program included wanting to gather stroke-related information and resources. Information sharing amongst participants and speakers was described as an important aspect of virtual CBO stroke programs, with participants confirming the significance of information sharing: “Both the weekly and monthly [meetings], you know there’s a lot of information that I’ve gained by listening to other people” (PV3, age 64, 6 years post-stroke). Another participant shared the importance of providing knowledge to individuals with stroke, stating, “People that have had a stroke have come from many walks of life. They don’t need things dumbed down, they just need to be given the knowledge” (PV1, age 64, 3 years post-stroke).

Participants also explained how the virtual CBO stroke programs provided an environment where they could connect with stroke experts: “…there are specialists that come on and talk about very specific things with regard to some of the after stroke issues that you might have that you’re not even aware of…” (PV7, age 69, 2 years post-stroke). The importance of having specialists and experts share stroke-related information in the virtual CBO stroke programs was highlighted by another participant, who spoke about gaining new perspectives, “Like you know you learn things like oh I could do this better, or you know, maybe I should try this, stuff like that” (PV5, age 39, 2 years post-stroke). Additionally, opportunities for connections to knowledge and information outside of immediate geographical location were highlighted as a benefit by a participant:

…having experts to talk to, and then having all different and varying opinions and information…Staying within my own community and trying to work through this network for me wasn’t going to work. I had to go outside of my community to a larger community to get help (PV7, age 69, 2 years post-stroke).

### Theme 2: perceived barriers to accessing and participating in virtual CBO stroke programs

The second theme describes participant perceptions of barriers to accessing and participating in virtual CBO stroke programs. Participant-perceived barriers were categorized into three subthemes: technological inequities (subtheme 2a), difficulties navigating technology (subtheme 2b), and impact of facilitator instruction (subtheme 2c).

#### Subtheme 2a: technological inequities

This subtheme describes participants’ descriptions of aspects of virtual CBO stroke programs that made it challenging for them or could make it challenging for others with stroke to access these programs. While all participants owned or had access to a computer, smartphone or tablet and Internet connectivity and were comfortable with technology use, participants touched on several factors that could reduce access to these programs for others with stroke. For instance, if someone with stroke did not have access to a computer/Internet, technical literacy, technical support, and technical experience, they would not be able to participate. PV6 recognized their privilege of technology ownership, “I’m lucky enough to have the, the computer technology at home” (PV6, age 56, 3 years post-stroke). Similarly, PV7 recognized that not all people with stroke would have this privilege: “I think that might be an issue for some people, is that they don’t have the technology. I have the technology… But some people don’t, and it can limit them” (PV7, age 69, 2 years post-stroke).

In addition to technology ownership, participants discussed the challenges they had experienced with setting up and using the platform that the virtual CBO program was delivered on. Participants indicated that the need for technical support, particularly assistance with logging on to the virtual platforms (e.g., Zoom), could be a major barrier for some people who had a stroke that may prevent their access to the program. While some participants could rely on their family or friends for technical support, they recognized that it was not always practical for them to rely on others: “…to have somebody there that would help me get on to these things, that would be the truth, but it’s not always practical” (PV1, age 64, 3 years post-stroke).

Finally, a lack of technical experience was recognized as another barrier that could limit access to virtual CBO stroke programs. PV6 acknowledged how previous experience with technology would benefit ease of access: “I’m very comfortable with Zoom because prior to the stroke happening, like this was our daily occurrence with meetings, so for me the technology was no problem, but some people were um challenged…” (PV6, age 56, 3 years post-stroke). Difficulties related to lack of previous experience with technology combined with stroke-related cognitive or communication challenges were illustrated in a comment by PV11, who discussed the challenges of navigating and understanding virtual platforms and highlighted a lack of technical literacy and expertise:

When I was there, I kind of didn’t like technology…I couldn’t talk and sometimes …it was really confusing with, like if you want to go to the internet, and they say that like there’s like a little screen saying something and I didn’t understand what’s going on, so I kind of like not. I didn’t like technology (PV11, age 33, 11 years post-stroke).

Participants highlighted that having prior access to and experience with computers and conferencing platforms and technical support influenced an individual’s ability to engage in virtual CBO stroke programs. Social support could also help facilitate access for those who experienced challenges after a stroke that may limit their access and participation in virtual CBO stroke programs.

#### Subtheme 2b: difficulties navigating technology

Participants described barriers to participating in virtual CBO programs involving issues using technology and the vulnerability of a virtual space. For example, a commonly cited barrier to meaningfully participating in these programs was difficulties using technology related to setting up the technology to allow for optimal viewing and engagement and muting and unmuting the speaker. These experiences were shared by PV7, who described challenges with learning how to set up the camera so that they could be seen in the viewfinder,

You know if you’re working on your phone and…you’re trying to manage Zoom and seeing, and, and where do you hold it, and how do you do it, your arm gets tired and where do you put it, and even on an iPad it can be sometimes a little hard (PV7, age 69, 2 years post-stroke).

A second commonly cited barrier to participation was the aspect of vulnerability in a virtual environment. General difficulties participating and engaging in virtual programs involved concerns about feeling uncomfortable about speaking within the group. PV8 stated, “…when you’re trying to speak openly, it’s not so comfortable to speak to strangers, and you don’t know who they are right, so it’s kind of awkward when you have that” (PV8, age 50, 23 years post-stroke). According to PV7, building “a deep-rooted connection with someone” in an online environment “will depend on what the other person on the other end wants” (PV7, age 69, 2 years post-stroke).

Other barriers present in the virtual environment included those applicable to individuals who might have difficulties with public speaking or the feeling of being over-exposed, as PV4 mentioned,

I think, in some cases…like because it’s it’s showing it showing your audio and your video sometimes, and this is the reason why we didn’t have video phones a lot earlier is because people didn’t want to be seen. So you know, sometimes, sometimes you know you’re not feeling very happy or whatever and you’d rather not (PV4, age 65, 6 years post-stroke).

#### Subtheme 2c: Impact of facilitator instruction

According to participants, their participation in virtual programs depended on the skills and effectiveness of the facilitator. For instance, PV11 shared an example of witnessing poor facilitation, which prevented program participants, particularly those with post-stroke communication challenges, from meaningfully participating in the virtual sessions: “Sometimes people wanted to talk, and I wanted to tell like they can go, but the person was the leader from the meeting and said next question so I’m like oh” (PV11, age 33, 11 years post-stroke). Participants also explained that a critical role of facilitators was that they had to manage and redirect some individuals who spoke for too long during the program, and if the conversation was not managed adequately, it prevented opportunities for others to engage in the discussion: “Some people like it, because they’re very talkative and they’ll just drone on, and they have no filter in their head to say oh well maybe I should shut up for a bit and let somebody else talk” (PV10, age 40, 2 years post-stroke).

Participants also identified difficulty receiving individualized instructions from program facilitators as a barrier to participation. In physical activity and exercise programs, individualized feedback or instruction from facilitators was crucial to engagement and continued participation, given that individuals with stroke have varying levels of function and challenges. One participant stated,

When you’re working in a group, you always work to the lowest common denominator. And with the stroke victims, you have every kind and every ability represented. I just feel that we’re doing the exercises, but all of us are doing them at your own ability. But there’s nobody pushing you…(PV1, age 64, 3 years post-stroke).

Another common barrier to participation reported by participants was the quality of facilitator communication and ability to lead the group effectively. Ineffective communication was most commonly noted by participants who participated in physical exercise classes led by students and experienced facilitators. One participant mentioned these feelings by saying,

People that have had a stroke have come from many walks of life. They don’t need things dumbed down. They just need to be given the knowledge. And I felt that he was a seasoned communicator and could deliver like he needed to deliver quickly, whereas students were kind of pussyfooting around the message (PV1, age 64, 3 years post-stroke).

PV7 also mentioned the difficulties that facilitators had managing technology, effectively creating barriers to participation for individuals with stroke, saying, “They would put up a presentation and… didn’t know how to expand the presentation, so that you can actually see, all you see is a tiny little box” (PV7, age 69, 2 years post-stroke).

### Theme 3: perceived facilitators to accessing and participating in virtual CBO stroke programs

The third theme describes the characteristics that participants identified as enabling their access (subtheme 3a) and continued participation (subtheme 3b, subtheme 3c) in virtual CBO stroke programs.

#### Subtheme 3a: remote access to virtual CBO stroke programs

Many participants cited the virtual CBO stroke programs’ remote nature as a facilitator to accessing virtual CBO stroke programs. While all participants in this study resided in an urban or suburban location, they noted that the ability for others to connect to the program from any location, including rural and remote areas, made the programs very accessible to people with stroke.

This is offered to you…and it’s- it’s offered to everyone, and you can go on and you, you can see what other people are experiencing across the whole country. And and so takes it out of your own little - now let’s say you live in a farm community where, where you don’t have the access right, so you have access (PV3, age 64, 6 years post-stroke).

Remote access to virtual CBO stroke programs was also discussed in terms of enabling continued engagement while on vacation or during travel: “Well the virtual program allows me to continue participating, you know, even though I’m in Florida” (PV3, age 64, 6 years post-stroke). PV3 reflected that if the programs were in-person, he would no longer be able to access the program until he returned home.

For participants unable to drive after stroke due to post-stroke impairments, removing transportation barriers with remote program access was discussed as an important facilitator. For instance, PV4 highlighted the very nature of virtual programs allowed his access to the programs because he did not drive and would otherwise not be able to access the program if it were in-person: “I can’t drive, so it’s limited me in that way, but that’s where Zoom-, Zoom can come in” (PV4, age 65, 6 years post-stroke). Moreover, participants explained that accessing the virtual stroke program without having to arrange transportation with friends and family was a critical benefit that facilitated their access. PV6 explained that this was important because she relied on her husband for transportation to multiple appointments, and the virtual program reduced her reliance on her husband: “I prefer online right now because it’s one less place my husband has to take me” (PV6, age 56, 3 years post-stroke). Other participants shared how remote virtual CBO stroke program access enabled energy conservation because of reduced physical demands to attend the sessions.

#### Subtheme 3b: enabling virtual platform features

Participants discussed how the functions of the virtual platforms enabled the participation of people with stroke in these virtual CBO stroke programs. Participants explained that virtual platforms, such as Zoom, provided various ways to communicate and participate in program sessions, such as through verbal communication or chat. They highlighted that multiple methods of communication were important as many people with stroke experience communication challenges. For instance, PV7 explained how the messaging function on the Zoom chat allowed participation from those who had memory impairments after a stroke, which could make it difficult to remember what was said during program sessions: “…having the answer come back to you is really important…then you get it in writing” (PV7, age 69, 2 years post-stroke). Participants also explained that other features of the virtual platforms, such as automatic virtual reminders about scheduled program sessions, were highlighted as a facilitator of engagement. PV7 shared their appreciation for program reminders: “And the reminders that automatically go into your calendar, so pops up on your phone, like yours popped up on my phone immediately, and then I’m like oh great yeah” (PV7, age 69, 2 years post-stroke). The ability to control how one participated in the virtual CBO stroke program was also discussed as a facilitator: “…you can go not on camera, and then you can participate” (PV11, age 33, 11 years post-stroke).

#### Subtheme 3c: program leader characteristics

Participation was facilitated in virtual CBO stroke programs through program leaders’ ability to communicate clearly with people who had experienced a stroke. PV7 discussed how the program leader helped her participate when first starting the program: “They would tell you what the program was going to be all about, giving you an agenda and an outline, and-, and then give you several different ways of logging in and participating” (PV7, age 69, 2 years post-stroke). Participants also identified the importance of the program leaders’ prior experience in facilitating virtual CBO stroke programs. PV3 shared, “…I mean, you get very competent people that know what questions to ask to get you to know to shape the conversation” (PV3, age 64, 6 years post-stroke).

### Theme 4: unmet needs during virtual CBO stroke programs

This fourth theme describes the unmet needs for in-person connection (subtheme 4a) and individualized support (subtheme 4b) due to the virtual CBO stroke programs’ format and structure.

#### Subtheme 4a: unmet needs for in-person connection

Participants frequently discussed their desire for increased connection with peers in the virtual CBO stroke program. Participants discussed that in the virtual program format, they were missing out on the connections that form through being physically together in person. PV11 shared this desire for in-person, physical connection: “…I can see the people, and you just want to hug them, but you can’t” (PV11, age 33, 11 years post-stroke). Other participants discussed how this lack of physical, in-person connection affected the quality of connection they could develop with other participants in a virtual program. PV3 expressed the different quality of connection with virtual and in-person opportunities: “I mean it’s not quite the same thing as sitting around coffees, you know, being online, and you know being distanced, so it’s you know you lose something in the connectedness that you have with the other people” (PV3, age 64, 6 years post-stroke).

#### Subtheme 4b: unmet need for individualized support

The participants discussed a lack of individualized support and instruction in the virtual CBO stroke programs. Desires for in-person feedback from the facilitator to provide reassurance of participants’ performance were commonly shared, particularly because people with stroke had diverse needs and impairments. PV1 discussed the need for more direct support from the program facilitator, saying: “I would try to get some in-person help, so that…I knew ‘are you doing it right,’ ‘are you doing it wrong,’ or ‘that’s okay’…if you tried a little harder, you could put your weight here (PV1, age 64, 3 years post-stroke). Similarly, participants discussed the unmet need for the facilitator to accurately perceive the participants’ emotions during the virtual program to provide appropriate support. PV8 said:

…it would have been more beneficial if it was in person because then the person – the instructor would be able to see your whole body instead of just your legs or whatever… That point of view it was kind of hard because they don’t see if you’re making a funny face or in pain – that kind of thing. From that point of view it makes a difference because when you are just focused on our legs [unintelligible] that’s all you see on the screen, so you can’t keep moving the screen all the time, so from that point of view, I would not recommend virtual because it’s not so easy to show somebody you’re in pain (PV8, age 50, 23 years post-stroke).

### Theme 5: suggestions and preferences for improving virtual CBO stroke programs

The fifth theme describes participant-identified suggestions and preferences for improving the current virtual CBO stroke programs, including suggestions for effective facilitation of virtual programs (subtheme 5a), virtual content preferences (subtheme 5b) and delivery format preferences (subtheme 5c). Based on the findings of this study, we have compiled a list of recommendations to optimize virtual CBO stroke programs (see Table 3).

**Table 3:** Virtual CBO stroke program recommendations

Target area of recommendation:	Practice recommendations for virtual CBO stroke programs:
**Session: Preferred program content**	-Include participants’ experiences of stroke in virtual program sessions -Tailor program content to each participant needs (for example, a few participants suggested including practical stroke-related education, navigating the healthcare system, applying for funding).
**Facilitation: Communication**	-Implement and enforce standards for communicating in program sessions, such as raising hands on Zoom and clear communication, to promote order and ease of sharing in the virtual setting -Incorporate frequent breaks during sessions to check in with participants -Provide participants with individualized support and feedback
**Facilitation: Training & rapport-building**	-Provide facilitators with ongoing opportunities to improve virtual facilitation skills -Explore how to optimize individualized feedback and staffing in a virtual group format
**Program structure: Delivery format**	-Offer multiple delivery methods for program engagement (i.e., hybrid delivery), including both in-person and virtual options for program sessions

#### Subtheme 5a: suggestions for effective facilitation of virtual programs

Participants provided suggestions to enhance the effectiveness of the virtual program facilitation. PV11 discussed the importance of including a method to promote order in virtual participant communication, such as allowing one participant to speak at a time:

​​I think if they have a question and then they will say who wants to- to speak and then raise your hand and then get a person to write things down that’s like 10 people, okay so 10 and then you cross the people out, then they talk and then they say anybody else… (PV11, age 33, 11 years post-stroke).

Other suggestions focused on improving facilitator communication, including monitoring and slowing communication speed. Multiple participants discussed how altering communication speed was important for people with post-stroke communication challenges to receive information meaningfully. A participant expressed this by saying, “the really important thing, at least on the aphasia front, is just being very patient and speaking very slowly” (PV12, age 58, 3 years post-stroke).

#### Subtheme 5b: virtual content preferences

Participants shared their preferences regarding the content of virtual program topics and learning materials. Many participants suggested that the virtual programs provided an effective context to engage in dialogue-based discussions about stroke and sharing stories of their stroke experience with PV3 sharing: “And they would talk about their stroke experience and how it changed their life and what challenges they’re facing going forward. Those, those times were very interesting. Great to listen to” (PV3, age 64, 6 years post-stroke). Conversely, PV6 identified preferences towards less personal sharing during the virtual program and more practical discussions about navigating life after a stroke:

I think something that would be also beneficial is like, even if it was a pre-recording on the how to navigate the system…like there’s different avenues or different routes, you have to go… Like what’s available…just the general stuff like I had no idea on how to apply for the like the benefits like to get the wheelchair and to apply for those programs was kind of just left up to you to navigate (PV6, age 56, 3 years post-stroke).

#### Subtheme 5c: delivery format preferences

Participants identified preferences for the delivery format of CBO stroke programs. Some participants (*n* = 4) had previously attended the in-person CBO stroke program(s) before COVID-19, while others (*n* = 8) had only participated in the virtual stroke programs either due to transportation or geographical limitations, having experienced a stroke after the programs transitioned to virtual delivery, the program was not previously being offered in-person, or they were unaware of the program. Of the twelve individuals interviewed, nine (75%) expressed interest in a hybrid delivery format citing a number of reasons, including increasing availability and variety of programs offered with PV10 sharing:

I’d prefer a hybrid model of it because I’m sure there’s different strokes for different folks, mind you, because, like myself most of the groups that I’ve…engaged with are not here in Ontario there’s not very much for Ontario- for young stroke survivors (PV10, age 40, 2 years post-stroke).

Other participants suggested an interest in hybrid delivery due to the options they provide for engagement. PV10 defined their preferences: “My version of hybrid is…being able to be in person and have people be able to view the-, the meeting online as well and participate in some fashion or another” (PV10, age 40, 2 years post-stroke). Hybrid modes of program deliver programs were similarly discussed by another participant for the choice hybrid offers in participation methods:

I’ve thought about this a lot. I think it’s good for a hybrid or have a personal group. Three of us can get together in person, and we bring our laptops, and everybody else Zoom in. Like it’s not one, it’s hybrid - so Zoom or in person (PV12, age 58, 3 years post-stroke).

In contrast, another participant preferred only an online format because it removed the need to mobilize in the community with PV6 sharing: “I prefer online right now for the - for those type of things just because it’s easier with - I have to have someone take me…we live in a two-level house and even to get outside, I have to climb stairs” (PV6, age 56, 3 years post-stroke).

## Discussion

This study describes the experiences of individuals with stroke in virtual CBO stroke programs. Five themes were identified: (1) motives for participating in virtual CBO stroke programs, including gaining peer connections, knowledge and information; (2) perceived barriers to accessing and participating in virtual CBO stroke programs, including technology inequities, difficulties navigating technology, and inadequate facilitation; (3) perceived facilitators to accessing and participating in virtual CBO stroke programs, including remote access, virtual platform features and program leader characteristics/skills; (4) unmet needs during virtual CBO stroke programs, including in-person connection and individualized support; and (5) suggestions and preferences for improving virtual CBO stroke programs, including program facilitation, content and format. Our study findings resonate with and extend findings from prior literature examining the delivery of new virtual CBO programs while offering insights into participant experiences to help inform future virtual CBO stroke program delivery.

A key insight of our study was that most participants identified the potential of hybrid program delivery formats (virtual and face-to-face components). Most participants in this study had easy access to the technology and were comfortable with using the technology, which may have suggested an increased willingness for virtual programming. Although most participants thought the idea of hybrid might be interesting, allowing them to get the benefits of both virtual and in-person programs, it is important to note that they had experienced this format and, therefore, additional research would be required on the implementation and impact of a hybrid model of delivery. Participants indicated that they joined virtual CBO stroke programs to build peer connections; this was unsurprising given that research has found that individuals experience elevated levels of loneliness after stroke [54], and the risk of depression after stroke, which is found associated with the stroke survivors’ self-efficacy in participation [55]. However, the usefulness of virtual stroke programs in promoting self-efficacy in participation and addressing their psychosocial needs warrants further research. Despite the benefits of virtual program delivery, such as increased flexibility in participant attendance, connecting with others whom one would not be able to otherwise, and eliminating the need for travel [30, 33], there remained a desire for increased peer connections through an in-person or hybrid connection that was a key aspect unfulfilled within the virtual CBO stroke program. However, such needs being unmet or not fully satisfied are not unique to virtual CBO programs, as prior literature has suggested that interpersonal communication in virtual environments differs from in-person interactions and may be hindered within virtual environments due to the absence of sensory communication (e.g., smell and touch), limited visual and direct bodily interactions [56–58]. Thus, a key consideration for CBOs is optimizing virtual CBO stroke programs to facilitate stronger connections with others. For instance, virtual CBO stroke programs tend to be delivered in a group-based format, and one study suggests that large sizes can reduce meaningful connections with others and perceptions of compassion within these programs [59].

Given participant interest, CBOs may consider hybrid program delivery models combining in-person and virtual delivery [60], based on continuing to deliver the content that works well in virtual formats virtually (e.g., stroke information/education) and delivering the aspects that some participants report may not entirely fulfill their needs in-person (e.g., meaningful peer connections) [61]. Hybrid programs may reduce excessive screen time, which has been associated with negative mental health outcomes (e.g., psychological problems, low emotional stability, and depression or anxiety [62]. However, it is unclear whether hybrid program delivery will be the answer, as hybrid models are still in their infancy, and optimal design and delivery formats remain understudied [61].

Another important finding from this study is the unmet need for individualized instruction within virtual CBO stroke programs. Since individuals have diverse impairments and challenges after a stroke (e.g., visual, physical, cognitive and/or communication) [63, 64], some participants in our study expressed the need for more support than currently provided, hoping to receive individualized instructions/feedback. Furthermore, at the organizational level, virtual CBO stroke programs may require additional facilitation skills or training to meet diverse participant needs. This is particularly important given that volunteers are a key resource in CBOs [65, 66], and participants indicated that students or volunteers may not be as skilled in facilitation as staff facilitators. We suggest facilitators, including volunteers and students, should be sufficiently trained to facilitate these sessions. Training in two types of skills may be required for volunteers/students to support virtual CBO stroke programs: content and technology. Regarding content, facilitators should know how to effectively communicate with individuals with stroke, as approximately 50% of people with stroke have stroke-related communication impairments [67–69]. This training may help enhance the quality of relationships and interactions with facilitators, which is essential to supporting meaningful participation within virtual CBO stroke programs [59]. Regarding technology, facilitators should know how to effectively engage and build relationships with participants in virtual environments. Key factors that will influence the success of these programs include the effective use of virtual platform features and effective facilitation [59]. Virtual platform features, such as the chat box and the ability to turn the video help on/off, minimize the impact of post-stroke challenges, such as fatigue and aphasia, on their participation. These skills were previously identified as necessary components of compassionate support in virtual peer support stroke programs [59].

Optimal training on facilitation skills for students and volunteers may support more effective and widespread delivery of virtual CBO programs, as demand for stroke programs will continue to rise with the increasing number of people living with stroke [70]. While there are no specific best practices for virtual CBO stroke programs, some best practices for virtual stroke rehabilitation may be relevant to these programs, such as assessing individualized needs [60]. This unmet need may be alleviated by assessing each individual’s needs.

As noted by participants, virtual CBO programs may help remove transportation barriers. This was a frequently identified benefit of virtual program delivery in our study, particularly for participants who cannot drive after a stroke or experience post-stroke fatigue. This finding is a significant benefit for individuals with stroke because stroke-related impairments can restrict safe driving capabilities [23]. Participants reported that they experienced less fatigue in virtual programs compared to in-person programs. Since post-stroke fatigue is experienced by up to 85% of people with stroke, this finding is significant [71]. Additionally, remote access to virtual CBO stroke programs facilitated participation for some participants in this study; however, since all resided in urban and suburban locations, this was not a top priority for these participants. Gray and colleagues [30] found that individuals who previously faced a geographical barrier to community programs may find increased ease of access with virtual program delivery. In another study from Taylor and colleagues [33] examining a community-based stroke program for individuals in Northwestern Ontario, participants reported enablement factors, including the ability to attend sessions without extensive travel time.

While the findings from this qualitative study highlight the benefits of virtual CBO stroke programs, participant experiences also reveal challenges similar to previous findings examining virtual program delivery involving technological inequity [33] and uncovering feelings of vulnerability in the virtual environment that warrant attention by CBOs [59]. Marginalized groups, including those with less education, limited English communication, speech difficulties, and certain racial backgrounds, are most at risk of having unreliable or unequal access to virtual technology [72]. Marginalized groups are also disproportionately affected by stroke [73], suggesting that the technological needs of those engaging in virtual CBO stroke programs should be considered and advocated for in the future of virtual CBO stroke programming. As these perspectives were not represented in the current study, future research should identify marginalized groups’ experiences of access and participation barriers and facilitators in virtual CBO stroke programs.

Additionally, a noteworthy barrier identified in this study was feeling vulnerable in a virtual environment, which resonates with prior research [59]. Prior stroke literature has cited barriers to virtual stroke programs, including difficulties expressing feelings and emotions through virtual formats [33]. Online communication removes subtle cues typically picked up during an in-person conversation, and virtual meetings with a group of people create new difficulties, like knowing how long to pause after someone speaks or when to speak up [74]. Study participants identified concerns about being uncomfortable speaking up within the group and feeling over-exposed due to having their video on. Bailenson [75] suggests that virtual video platforms require excessive close-up eye gaze depending on the size and layout of video screens and involve increased self-evaluation from staring at a video of oneself. Future research is likely needed to delve further into vulnerability and over-exposure in virtual settings, particularly how these aspects affect individuals with stroke and their participation in virtual CBO stroke programs.

### Limitations and strengths

The first limitation of this study is that participants were recruited primarily through two CBOs. While participants attended multiple types of virtual stroke programs, the perspectives of individuals attending programs from other CBOs may not be adequately captured. There may also be observer bias in participant responses due to participants attending additional webinars and chat-boards, which should be considered when interpreting our results. Second, we required participants to attend a minimum of one session of a virtual CBO stroke program but did not collect information on the program name, the extent to which the programs were tailored to an individual’s needs or total number of virtual CBO program sessions participants attended. This information could have helped further contextualize participants’ experiences in virtual CBO stroke programs. For instance, if participants had only a single session, they may have provided less comprehensive insights about participation facilitators but may have offered more insights into access and participation barriers. Third, most participants had at least a college-level education and were comfortable using technology. These findings may not resonate with individuals with lower education levels or who are less comfortable with technology. Fourth, these interviews were conducted between January to February 2022; the COVID-19 pandemic required a rapid shift to virtual stroke program delivery. All study participants accessed virtual CBO stroke programs out of necessity for program continuation/attendance, regardless of being ready for such a transition. Some CBO stroke programs may have transitioned to in-person or hybrid delivery in the months and years following this data collection period. Further research may be needed to fully understand the barriers to accessing virtual stroke programs when there is an option to participate in person. Fifth, while we attempted to reduce the risk of possible self-assessment bias or recall bias in patients’ responses to the questions by asking participants to share examples during the interviews, this should be considered when interpreting our results. Finally, while our study sample included individuals of a large age range (32–69 years old) and at various stages poststroke (2–23 years post-stroke), which may have allowed us to gather insights into differences in experiences based on life stage and stage of recovery. However, since we did not collect information on participants’ clinical characteristics at the beginning of the programs, we were unable to compare participant experiences, and this reduces the generalizability of our results.

## Conclusions

This qualitative study provided valuable insights into the experiences of twelve individuals who experienced stroke and participated in virtual CBO stroke programs across Canada. Motivations for joining virtual stroke programs and barriers and facilitators to accessing and participating in these programs are identified. Participants also indicated unmet needs during virtual programs relating to in-person connection and individualized support. Suggestions to improve virtual CBO stroke programs identified in this study may help optimize program development and delivery.

### Electronic supplementary material

Below is the link to the electronic supplementary material.

Supplementary Material 1

## Data Availability

The datasets generated and/or analysed during the current study are not publicly available due ethical and privacy restrictions but are available from the corresponding author on reasonable request.

## Abbreviations

CBOs: Community-based organizations
CBO: Community-based organization
COVID-19: coronavirus disease of 2019
