# Supplementary material for: Exploring the experiences of adults with stroke in virtual community-based stroke programs: a qualitative descriptive study
Source: BMC Health Serv Res. 2024 May 7;24:600. doi: 10.1186/s12913-024-11043-7 (PMC11077787; doi:10.1186/s12913-024-11043-7)
Supplement: Supplementary file 1 — Supplementary Material 1 [file 12913_2024_11043_MOESM1_ESM.docx]

**Supplementary material A: Draft interview guide questions for virtual program users**

1. What online stroke programs did you participate in?

2. Please tell me what it was like to participate in this online program.

- - What were the reasons you joined this program?
  - What did you hope to get from this online program?
  - How many sessions did you attend?
  - How did you hear about the online program?
  - How was it to communicate with the program leader?

1. What was it like being a participant in this program?
   - How is this program different from the in-person program?
   - How is it similar to the in-person program?
   - How did you connect to the program?
   - Who helped you connect?
   - How was it participating in the program?
   - How were program instructions/education delivered? Did you have any difficulty understanding the program instructions/education?
2. What was the first day in this online program like?
   - Was it hard to join?
   - Did you need any help connecting?
   - What resources did you need to connect?
   - What were your thoughts/feelings?

4. What made it easier to participate in this online program?

1. What made it hard to participate in this online program?
   - How can this program be easier to use for other people with stroke?

6. Did you complete the entire online program?

· If no, please explain why not.

· What would have helped you complete the program?

1. Did you ever attend this program in-person?
   - If yes, what was the in-person program like?
   - How did the in-person program differ from the online program?
   - What format (in-person or virtual) do you prefer and why?
   - How did the virtual program benefit you? Specific probes: Mood, physical or psychosocial wellbeing, feelings of social isolation, knowledge, attitudes, behaviour
2. Would you recommend this program to others with stroke?
3. How do you prefer this program to be delivered in the future (e.g. virtual or in-person)?
